# Triazine-cored polymeric vectors for antisense oligonucleotide delivery in vitro and in vivo

**DOI:** 10.1186/s12951-020-0586-8

**Published:** 2020-02-18

**Authors:** Mingxing Wang, Bo Wu, Jason D. Tucker, Sapana N. Shah, Peijuan Lu, Qilong Lu

**Affiliations:** McColl-Lockwood Laboratory for Muscular Dystrophy Research, Carolinas Medical Center, 1000 Blythe Blvd., Charlotte, NC 28231 USA

**Keywords:** Triazine, Amphiphilic cationic polymers, Antisense delivery, Exon-skipping, Muscular dystrophy

## Abstract

**Background:**

The polymer-based drug/gene delivery is promising for the treatment of inherent or acquire disease, because of the polymer’s structural flexibility, larger capacity for therapeutic agent, low host immunogenicity and less cost. Antisense therapy is an approach to fighting genetic disorders or infections using antisense oligonucleotides (AOs). Unfortunately, the naked AOs showed low therapeutic efficacy in vivo and in clinical trial due to their poor cellular uptake and fast clearance in bloodstream. In this study, a series of triazine-cored amphiphilic polymers (TAPs) were investigated for their potential to enhance delivery of AOs, 2′-*O*-methyl phosphorothioate RNA (2′-OMePS) and phosphorodiamidate morpholino oligomer (PMO) both in vitro and in vivo.

**Results:**

TAPs significantly enhanced AO-induced exon-skipping in a GFP reporter-based myoblast and myotube culture system, and observed cytotoxicity of the TAPs were lower than Endoporter, Lipofectamine-2000 or PEI 25K. Application of optimized formulations of TAPs with AO targeted to dystrophin exon 23 demonstrated a significant increase in exon-skipping efficiency in dystrophic *mdx* mice. The best ones for PMO and 2′-OMePS delivery have reached to 11-, 15-fold compared with the AO only in *mdx* mice, respectively.

**Conclusion:**

The study of triazine-cored amphiphilic polymers for AO delivery in vitro and in *mdx* mice indicated that the carrier’s performances are related to the molecular size, compositions and hydrophilic-lipophilic balance (HLB) of the polymers, as well as the AO’s structure. Improved exon-skipping efficiency of AOs observed in vitro and *in mdx mice* accompanied with low cytotoxicity demonstrated TAP polymers are potentials as safe and effective delivery carrier for gene/drug delivery.

## Introduction

Antisense therapy is an approach to fighting genetic disorders or infections using short DNA-like molecules called antisense oligonucleotides (AOs). Duchenne muscular dystrophy (DMD) is an X-linked, lethal neuromuscular disorder caused by mutations in the dystrophin gene affecting 1 in approximately 5000 males at birth [1]. It is the most common childhood form of muscular dystrophy (MD) and around 50% of all MD cases, and the patients usually die in their late 20s [2, 3]. Fundamental treatments of DMD requires either correction or replacement of the mutated gene to restore function. In recent years, AO-mediated exon-skipping has been a promising therapy for treating DMD by facilitating “Skipping” of specific dystrophin gene exon(s), thus to correct specific genetic mutations and restore production of dystrophin protein [4–13]. Among the several AO macromolecules, 2′ *O*-methyl-phosphorothioate RNA (2′-OMePS) and phosphorodiamidate morpholino oligomers (PMO) have been deeply studied in clinical trials. The 2′-OMePS is resistant to nuclease degradation and relatively stable in biological systems compared with natural DNA and RNA, but the presence of negatively charged phosphate groups may restrict their overall uptake [14, 15], additionally the toxicity has limited its ceiling dose for clinic. Meanwhile, PMO, is a neutral molecule under physiological condition, having the deoxyribose rings replaced with morpholino linked through phosphorodiamidate intersubunits, exhibiting excellent stability and lower toxicity compared with other counterparts [16, 17]. However, the relatively charge-neutral nature of PMOs associated with poor cellular uptake and fast clearance in bloodstream has been identified as a major obstacle to delivery efficacy. These impediments together suggest that an imperative challenge in AO exon-skipping is to improve delivery performance with minimizing toxicity [5, 18, 19].

To improve delivery efficiency, chemically modification has been studied, such as: (1) Arginine-rich cell penetrating peptides or octaguanidine dendrimer have been conjugated into PMO, and substantial improvement in delivery has been reported in *mdx* mice, leading to near normal levels of dystrophin expression in body-wide muscles [4, 9–11]. (2) Peptide modified 2′-OMePS improved uptake in skeletal and cardiac muscle [20]. However, the densely packed positive charges are associated with higher toxicity, making further clinical application difficulty [11]. Furthermore, the complicated synthesis and purification in covalent modification make it more expensive, and potential peptide-related immune responses could prevent repeated administration.

The polymer-based drug/gene delivery is promising for the treatment of inherent or acquire disease, because of the polymer’s structural flexibility, larger capacity for therapeutic agent, low host immunogenicity and less cost than viral one [21–24]. Amphiphilic polymers developed for gene delivery by our group and others have demonstrated enhanced gene transfection in skeletal muscles [25–35]. Recently, a set of triazine-cored amphiphilic polymers (TAPs) constructed from Jeffamine M series and low molecular weight polyethyleneimine (LPEI) were prepared by combinatorial chemistry and characterized for pDNA delivery in vitro and in vivo [34], which can condense DNA efficiently with nanosized particles below 200 nm at the weight ratio 5 of polymer/pDNA, being stable in the presence of serum. The introduction of amphiphilic Jeffamine led to a significant increase in the cellular uptake of complexes with higher transfection efficiency in CHO, C2C12, and HSkMC cell lines, but without toxicity increasing against parent LPEI. The best polymer-formulated pDNA delivery produced transgene expression efficiency 6-, 29-fold of PEI 25k-mediated in vitro and in *mdx* mice, respectively.

Motived by the promising performances of TAPs for pDNA delivery, we investigated them for AO delivery in vitro and in dystrophic *mdx* mice in this study. The results demonstrated that TAPs obviously improved AO-induced exon-skipping efficiency compared with Lipofectamine-2000 (LF-2k) or Endoporter (a commercially available reagent for PMO delivery) in vitro, and significantly enhanced dystrophin expression with the AO targeting the mutated mouse dystrophin exon 23 achieved also when administered in *mdx* mice. Higher efficiency and less toxicity indicated the potential of the TAP polymers as AO delivery vector for the treatment of DMD or other genetic diseases.

## Materials and methods

### Materials

Dulbecco’s modified Eagle’s medium (DMEM), penicillin–streptomycin, fetal bovine serum (FBS), l-glutamine and HEPES [4-(2-hydroxyethyl)-1-piperazineethanesulfonic acid] buffer solution (1 M) were purchased from Thermo Fisher Scientific (Waltham, MA, USA). 3-(4,5-Dimethylthiazol-2-yl)-5-(3-carboxymethoxyphenyl)-2-(4-sulfophenyl)-2*H*-tetrazolium (MTS) was bought from BioVision Inc. (Milpitas, CA, USA). Phosphorodiamidate morpholino oligomer PMOE50 (5′-GGGATCCAGTATACTTACAGGCTCC-3′) targeting human dystrophin gene exon 50, PMOE23 (5′-GGCCAAACCTCGGCTTACCTGAAAT-3′) targeting mouse dystrophin gene exon 23 and Endo-porter were purchased from Gene Tools (Philomath, OR, USA). An arbitrary single-stranded 20-mer deoxyoligonucleotide with the sequence 5′-GGCCAAACCTCGGCTTACCT-3′ (phosphodiester), for the physicochemical study of polymer-oligonucleotide polyplex. AOs modified by 2′-O-methylation and phosphorthioation 2′-OMePSE50 (5′-GGGAUCCAGUAUACUUACAGGCUCC-3′) targeting human dystrophin gene exon 50, 2′-OMePSE23 (5′-GGCCAAACCUCGGCUUACCU-3′) targeting mouse dystrophin gene exon 23 used for delivery in vitro and in vivo were purchased from GenScript (Piscataway, NJ, USA). Other chemicals were obtained from Sigma-Aldrich Co. (St Louis, MO, USA) unless otherwise stated. The TAPs were prepared previously by us [34].

### Cell viability assay

Cytotoxicity was evaluated in C2C12E50 cell line using the MTS assay [30–35]. The relative cell viability was calculated by: $\left( {A_{\text{treated}} - A_{\text{background}} } \right)\, \times \, 100/\left( {A_{\text{control}} - A_{\text{background}} } \right),$ the absorbance (***A***) was measured at 570 nm using a Tecan 500 Plate reader (Tecan US, Inc, Morrisville, NC, USA) to obtain the metabolic activity of the cell. Untreated cells were taken as controls with 100% viability and wells without cells as blanks, and all viability assay was carried out in triplicate.

### In vitro transfection

The C2C12E50 myoblast and C2C12E23 differentiated cells lines expressing the reporter GFP was used in this study. The expression of GFP was controlled by the effective skipping of the inserted human dystrophin exon 50 sequence (hDysE50), mouse dystrophin exon 23 sequence (mDysE23), respectively [27].

#### C2C12E50

The C2C12E50 cell line was maintained in 10% FBS-DMEM in a humidified 10% CO_2_ incubator at 37 ℃. About 5 × 10^4^ C2C12E50 cells per well in 500 µL medium was seeded and allowed to grow until a confluence of 70%. Cell culture medium was replaced before addition of polymer/AO formulation with varying ratios. PEI 25K, LF-2k and Endoporter used as comparisons. Transfection efficiencies indicated by GFP production were recorded after 4-day incubation with the Olympus IX71 fluorescent microscope and digital images taken with the DP Controller and DP Manager software (Olympus America Inc., Centre Valley, PA, USA). Transfection efficiency was also examined quantitatively using fluorescence-activated cell sorting (FACS) Calibur flow cytometer (BD, Franklin Lakes, NJ, USA).

#### C2C12E23

The cell culture and delivery protocol are the same as in C2C12E50, the images were taken, and cell collected after 6-day treatment.

### In vivo delivery

This study was carried out in strict accordance with the National Institutes of Health Guide for the Care and Use of Laboratory Animals. The protocols were approved by the Institutional Animal Care and Use Committee (IACUC), Carolinas Medical Center (Breeding protocol 10-13-07A; Experimental protocol 10-13-08A). The Mice were free of standard pathogens. All injections were performed under isoflurane anesthesia, and all efforts were made to minimize suffering [10, 11, 27].

#### Animals and injections

Dystrophic *mdx* mice (C57BL/10 as genetic background) aged 4–5 weeks were used for in vivo testing [5 mice per group, mixed male/female (m/f), 3 m + 2f or 2 m + 3f in the test and control groups] unless otherwise stated. Mice were killed by CO_2_ inhalation at desired time points, and muscles and other tissues were snap-frozen in liquid nitrogen cooled isopentane and stored at − 80 ℃. The 2′-OMePSE23 (5′-GGCCAAACCUCGGCUUACCU-3′) and PMOE23 (5′-GGCCAAACCTCGGCTTACCTGAAAT-3′) targeting the boundary sequences of exon and intron 23 of the mouse dystrophin gene were used. For intramuscular (i.m.) injections, 5 µg 2′-OMePSE23 or 2 µg PMOE23 with or without polymer was used in 40 µL saline for each tibialis anterior (TA) muscle. For intravenous (i.v.) injection, 1 mg PMOE23 with or without polymer used in 100 µL saline. The muscles were examined 2 weeks later and all of tissues were processed with the operator blinded to the treatment groups.

#### Immunohistochemistry and histology

Serial sections of 6 µm were cut from the treated mice muscles. The sections were stained with a rabbit polyclonal antibody P7 for the detection of dystrophin protein as reported previously [17, 20, 36–38]. Polyclonal antibodies were detected by goat anti-rabbit IgG Alexa 594 (Invitrogen, Carlsbad, CA, USA). The counting number of dystrophin-positive fibers in a single cross-section was addressed using the Olympus BX51 fluorescent microscope (Olympus America Inc., Centre Valley, PA, USA). Section were also stained with hematoxylin and eosin (H&E) for histological assessment.

#### RT-PCR for in vivo samples

Total RNA was extracted from the muscle after dissection, and 100 ng of RNA template was used for a 50 µL RT-PCR with the Stratascript One-Tube RT-PCR System (Stratagene, Santa Clara, CA). Ex20Fo 5′-CAGAATTCTGCCAATTGCTGAG-3′ and Ex26Ro 5′-TTCTTCAGCTTGTGTCATCC-3′ for amplification of mRNA from exons 20 to 26. The intensity of the bands obtained from the treated *mdx* mice muscles was measured and compared with that from normal muscles of C57BL/6 mice by ImageJ software version 1.42 (National Institutes of Health, Bethesda, MD, USA), and percentage of exon skipping was calculated with the intensity of the two bands representing both exon 23 unskipped and skipped as 100%. Unskipped band included exon 23 is 1093 bp, skipped band without exon 23 is 880 bp.

### Transmission electron microscopy

The polymer/AO polyplex solution containing 1 µg of AO was prepared at a designated weight ratio in 100 µL 0.9% saline and analyzed using transmission electron microscopy (TEM; JEM-1400Plus TEM by JEOL USA, Inc.) with AMT-XR80S-B wide angle side Mount 8MPixel, CCD Camera. The samples were prepared using negative staining with 1% phosphotungstic acid [30–35].

### Statistical analysis

All the data was expressed as mean ± SD, and the data were analyzed using Two-tailed *t*-test with a value of *p* ≤ 0.05 being considered statistically significant.

## Results and discussion

### Synthesis and characterization of triazine-cored amphiphilic polymers (TAPs)

The synthesis and characterization of TAPs have been reported in our previous study [34]. The structure and code of Jeffamine M series, LPEI, and corresponding structures of the TAPs are presented as supplementary in Additional file 1: Figure S1 and Table S1, respectively.

### Cytotoxicity

**Fig. 1:**
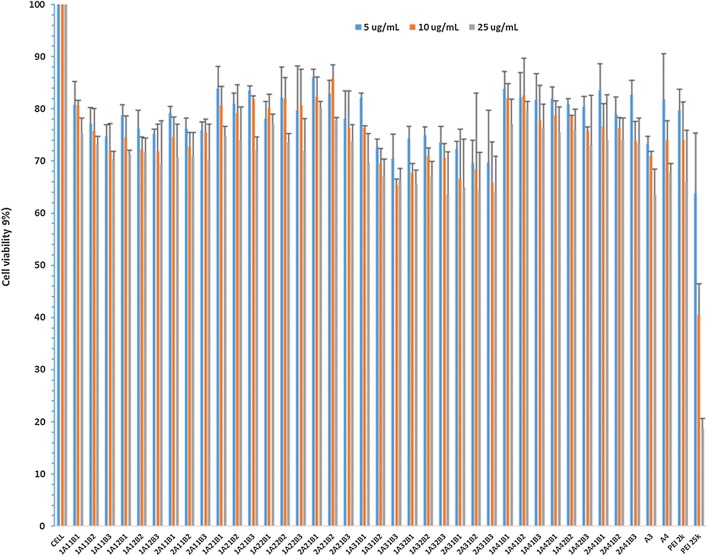
Cell viability of C2C12E50 myoblasts after treatment with TAPs at 3 doses (5, 10, 25 µg/mL; PEI 25k as control) determined by MTS assay. Cells were seeded in 96-well plate at an initial density of 1 × 10^4^ cells/well in 0.2 mL growth media. The results are presented as the mean ± SD in triplicate

The cytotoxicity of TAPs in C2C12E50 cells was evaluated by MTS assay (Fig. 1). PEI cytotoxicity was molecular size-dependent with higher Mw PEI exhibiting higher toxicity. The PEI 25k showed high cytotoxicity with cell viability around 20%; while LPEI 2k gave much lower toxicity with over 60% cell alive at the dose of 25 µg/mL, which indicated that LPEI (0.8 k, 1.2 k and 2.0 k) is much lower toxicity than the high Mw PEI 25k [30–35]. Cell viability with all of the TAPs at the dose of 25 µg/mL was similar to or a little higher than that of their parental LPEIs, and all showed lower toxicity than PEI 25k at the lowest dose of 5 µg/mL. The cytotoxicity under high dose of 25 µg/mL was in the order from higher to lower: A_3_ > A_4_; 1A_1_1B_3_ > 1A_1_1B_2_ > 1A_1_1B_1_; 1A_3_1B_3_ ≥ 1A_1_1B_3_ > 1A_4_1B_3_ ≥ 1A_2_1B_3_; 2A_3_1B_3_ ≥ 1A_3_2B_3_ > 1A_3_1B_3_. The level of toxicity was associated with PEI content and HLB of the TAPs. The lower HLB the molecule has, the more hydrophobic it is. The HLB of the A series is in the order A2 > A4 > A3 > A1. The PEI size is in the order: B3 (PEI 2k) > B2 (PEI1.2k) > B1 (PEI 0.8k). TAPs with more and/or bigger size PEI and more hydrophobic showed relatively elevated toxicity. Toxicity of all polymers was dose-dependent, but the levels of toxicity with TAPs were consistently much lower when compared with PEI 25k at the same dose. This improved cyto-compatibility is probably attributed to the low-toxicity of the building blocks, and the possible steric hindrance of Jeffamines reducing positive charge density of PEI, thereby leading to a decrease of the average protonation constant of the polymers [39].

### Evaluation of 2′-OMePS and PMO delivery in vitro

In this study, the C2C12E50 myoblast and C2C12E23 differentiated cells expressing the reporter GFP upon differentiation were used for in vitro study. The expression of GFP in the cells were interrupted by the insertion of the human dystrophin exon 50 (hDyse50), or mouse dystrophin exon 23 (mDysE23) within the GFP coding sequence. Restoration of GFP expression relied on the targeted removal of exon(s) through exon-skipping by AOs [40, 41].

#### Negatively charged 2′-OMePS delivery in C2C12E50 myoblast cell

**Fig. 2:**
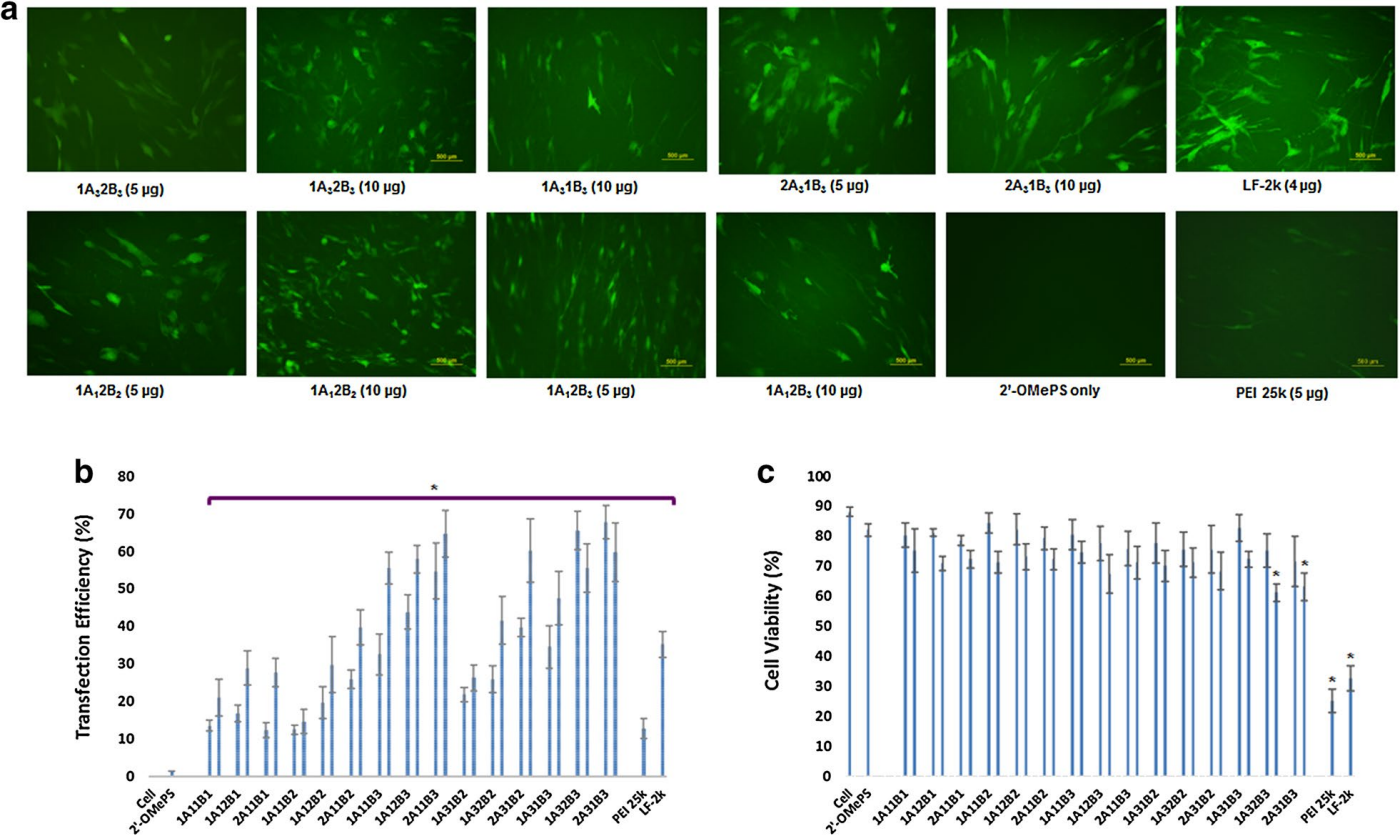
Delivery efficiency and toxicity of TAP/2′-OMePS E50 complexes in a C2C12E50 cell line determined by fluorescence microscopy and fluorescence-activated cell sorting (FACS) analysis. **a** Representative fluorescence images of 2′-OMePS E50-induced exon-skipping in C2C12E50 cell line. (The images were taken 4-day after treatment. Original magnification ×200; scale bar 500 µm). **b** Statistical date of TE of 2′-OMePS E50 formulated with TAP (Student’s *t*-test, *p ≤ 0.05 compared with 2′-OMePS E50 only). **c** Cell viability (Student’s *t*-test, *p ≤ 0.05 compared with untreated cell as control). In this test, 2 µg 2′-OMePS E50 was formulated with TAPs (5 and 10 µg), and PEI 25 (5 µg), LF-2k (4 µg) formulated as comparison in 0.5 mL 10% FBS-DMEM medium, respectively. The results are presented as the mean ± SD in triplicate

We first examined the effect of TAPs on exon-skipping of 2′-OMePS in the C2C12E50 cell line. The AO sequence (5′-GGGAUCCAGUAUACUUACAGGCUCC-3′) targeting the inserted human dystrophin exon 50 within the GFP coding region was used. The cells were treated with the 2′-OMePSE50 at a fixed amount (4 µg/mL) formulated with each TAP at different dosage. Transfection efficiency (TE) of formulated 2′-OMePSE50 was visualized under fluorescence microscopy analysis after 4-day delivery. The delivery efficiency of TAPs (at the dose of 10 to 20 µg/mL) formulated 2′-OMePS were measured quantitatively by flow cytometry (Fig. 2). Delivery efficiency was observed over 50% with 1A_1_1B_3_, 1A_1_2B_3_, 2A_1_1B_3_, 2A_3_1B_2_, 1A_3_2B_3_ and 2A_3_1B_3_ and over 65% with 2A_1_1B_3_, 1A_3_2B_3_ and 2A_3_1B_3_. In contrast, 2′-OMePS alone, and 2′-OMePS formulated with PEI 25k or LF-2k exhibited around 1.5%, 12.6% and 35.2% GFP positive cells, respectively, at the optimal concentrations. The 2′-OMePS with the best TAP formulation achieved the highest efficiency with over 44-fold of that achieved with 2′-OMePS alone. Clearly, the results suggest that a combination of amphiphilic part and cationic ingredients together are preferred as carrier to enhance delivery efficiency of 2′-OMePS. In agreement with the viability results from polymer alone, cytotoxicity of the TAP/2′-OMePS complexes was dramatically lower than that of PEI 25k or LF-2k formulated 2′-OMePS complexes. These results are in line with that for pDNA delivery [34]: the more hydrophobic A_1_/A_3_-based (lower HLB) ones showed more effective than the more hydrophilic A_2_/A_4_-based TAPs (higher HLB, all were below 10%, data not given here), and more positive ones (2B composed TAPs; B3 composed > B2 composed > B1 composed TAPs, correspondingly) demonstrated more effective also, further demonstrated the complicate interactions between TAP and 2′-OMePS including charge and hydrophobic interaction.

The transduction and cytotoxicity were further exemplified by staining with Propidium iodide (PI) and Hoechst 33342 after 6-day transfection (Additional file 1: Figure S2). The delivery efficiency of 2A_3_1B_3_ formulated 2′-OMePS was much higher than LF-2k mediated one at the optimum condition based on our early study [32, 34, 35], while the toxicity was much lower than LF-2k, even 2A_3_1B_3_′s dose was 2.5 times of LF-2k. This further indicates the advantage of TAPs as carrier of oligonucleotide delivery against LF-2k, which is probably due to TAP’s amphiphilicity and moderate positive charges.

#### Uncharged PMO delivery in C2C12E50 myoblast cell

**Fig. 3:**
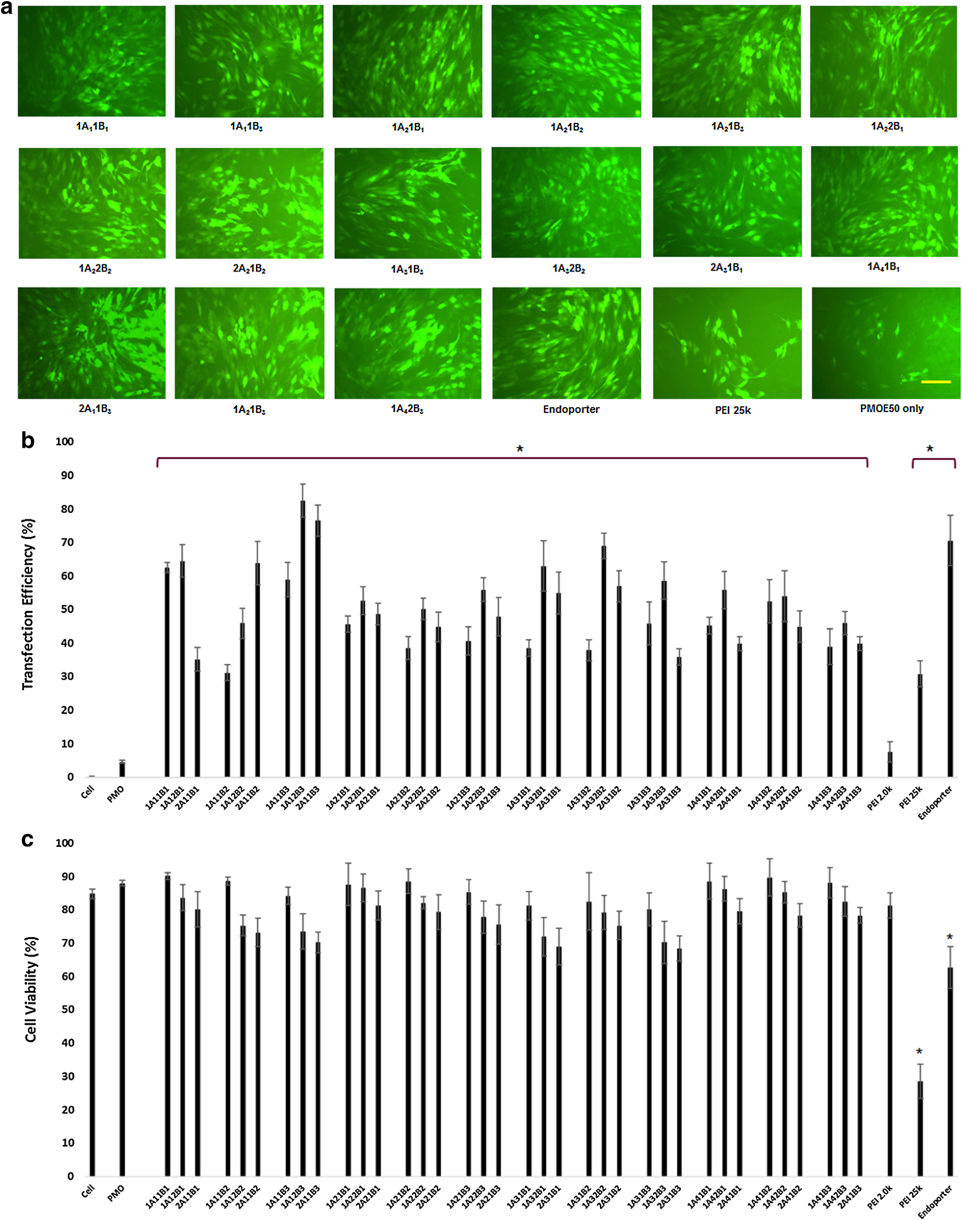
Delivery efficiency and toxicity of TAP/PMOE50 complexes in a C2C12E50 cell line determined by fluorescence microscopy and flow cytometry. **a** Representative fluorescence images of PMOE50-induced exon-skipping in C2C12E50 cell line. (The images were taken 4-day after treatment. Original magnification ×200; scale bar 200 µm). **b** Statistical date of TE of PMOE50 formulated with TAP (Student’s *t*-test, *p ≤ 0.05 compared with PMO only). **c** Cell viability (Student’s *t*-test, *p ≤ 0.05 compared with untreated cell as control). In this test, 5 µg PMOE50 was formulated with TAPs (10 µg), and PEI 25 (5 µg), Endoporter (5 µg) formulated as comparison in 0.5 mL 10% FBS-DMEM medium, respectively. The results are presented as the mean ± SD in triplicate

C2C12E50 cells were treated with 10 µg/mL PMOE50 (5′-AACTTCCTCTTTAACAGAAAAGCATAC-3′) formulated with each polymer at two doses (10, 20 µg/mL) in 0.5 mL 10% FBS-DMEM, and the TE was determined by fluorescence microscopy and FACS after 4-day treatment as for 2′-OMePS E50 (Fig. 3). PMOE50 formulated with polymer 1A_1_1B_1_, 1A_1_2B_1_, 2A_1_1B_2_, 1A_1_1B_3_, 1A_1_2B_3_, 2A_1_1B_3_, and 1A_3_2B_3_ reached TE over 60%, and the better ones were hit by 1A_1_2B_3_ and 2A_1_1B_3_ close to or over 80%, comparing with Endoporter-mediated reaching 70% and PMO alone with around 4%. These levels of GFP expression of best TAP-mediated PMO were approximately 20-fold greater than that achieved by PMO alone. Cytotoxicity of the amphiphilic TAPs formulated PMO at this dose remained low, with more than 70% of cell surviving rate for all TAPs. In contrast, PEI 25K or Endoporter kept around 30% and 65% cell alive at a dose of 10 µg/mL, respectively. On the other hand, the more hydrophilic A_2_/A_4_-based TAPs gave much better efficacy for PMO delivery compared with that for 2′-OMePS or pDNA [30–35], although a little lower than the more hydrophobic A_1_/A_3_-based TAPs. The overall performances both in efficiency and cytotoxicity are related to the size and HLB of the TAPs. This is directed by much higher exon-skipping efficiency obtained with 1A_1_1B_1_, 1A_1_2B_1_, 2A_1_1B_2_, 1A_1_1B_3_, 1A_1_2B_3_, 2A_1_1B_3_, and 1A_3_2B_3_, which indicated the complicated relationship between the polymer composition and delivery activity to delivery cargos: (1) The amphiphilic nature of TAPs are important to complex with delivery cargos to form stable and effective particles; (2) The TAPs with moderate size and bigger or more PEI content are preferred as negatively charged pDNA or 2′-OMePS delivery carrier; (3) The positive charge of polymers for delivering uncharged PMO is not so imperative as delivering negatively charged pDNA or oligonucleotides, but moderate charged amphiphilic TAPs improved the size-matched PMO delivery efficiency probably through TAP/PMO complex particles formed by hydrophobic–hydrophobic interaction and additional hydrogen-bond with dispersed positive surface, leading to TAP/PMO complex more flexible and more preferable to improve cellular uptake.

#### PMO delivery in C2C12E23 differentiated cell

**Fig. 4:**
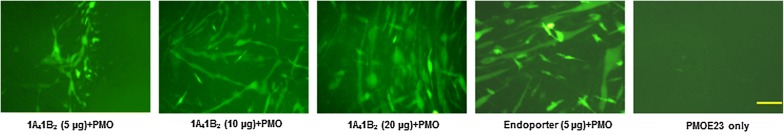
Green fluorescent protein expression induced by PMOE23 (5 μg) formulated with 1A_4_1B_2_ dose-dependent in C2C12E23 cells in 0.5 mL of 10% FBS-DMEM after 6-day treatment. Original magnification ×200; scale bar 200 µm

To evaluate the delivery potential of the TAPs for PMO exon-skipping in muscle fibers, the TAPs were further tested in the mouse dystrophin exon 23 reporter C2C12 cell (C2C12E23). The C2C12E23 reporter construct uses a muscle creatine kinase (MCK) promoter to drive the GFP expression, thus allowing us to evaluate more cell-type exon-skipping of AOs in differentiating or differentiated myotubes [41]. Cells reaching around 70% confluence were incubated in 10% FBS media for 2 days and then treated with TAPs formulated PMOE23. The PMOE23 (5′-GGCCAAACCTCGGCTTACCTGAAAT-3′) targeting the boundary sequences of exon and intron 23 of mouse dystrophin gene was used here. We exemplified the 1A_4_1B_2_ with more hydrophilic nature and better performance in C2C12E50 cell line as model at three doses (10, 20 and 40 µg/mL) formulated PMOE23 (10 µg/mL) per our previous study, and the TE was visualized by fluorescence microscopy after 6-day treatment. The results demonstrated that the 1A_4_1B_2_ enhanced exon-skipping of PMO as compared to PMO alone, the dose-dependent efficacy to differentiated cells was observed, and no toxicity detected under the tested dosage as illustrated images in Fig. 4. The delivery efficiency at the dosage of 20 or 40 µg/mL is comparable or higher than Endoporter achieved.

Our study showed A2/A4-based TAPs with lower efficiency for pDNA and 2′-OMePS delivery in cell culture and in vivo delivery [30–35]. However, the same TAPs were highly effective for PMO delivery with enhanced exon-skipping. The reason for such an apparent difference in TAP-mediated delivery for pDNA, 2′-OMePS and PMO are unclear, but the differences in chemical nature, molecular size and delivery mechanism existing among these three therapeutic cargos are mostly responsible. The negatively charged pDNA or 2′-OMePS requires more positively charged polymeric vector to condense a complex stable for effective delivery, leading to the polymer/cargo polyplex’s particle size and surface charge under same weight ratio is completely different. Instead, TAPs and uncharged PMO may form complex through primarily hydrophobic interactions, and may assemble high order superstructure. This has been demonstrated in our early study that the lipophilic interaction between amphiphilic polymer and uncharged PMO might therefore be one of the critical factors to enhance PMO delivery [30, 31, 33, 35]. In addition, positively charged groups within the TAPs might not only play a key role for the interaction with PMO through hydrogen-bonds, but it could afford the polyplex particles in a physiological environment a longer circulation half-life also than PMO alone. This may well lead to a higher serum tolerance and improvement in the uptake of PMO through the vasculature and cell membrane, therefore more effective delivery of PMO into muscles.

### Transmission electron microscopy (TEM)

**Fig. 5:**
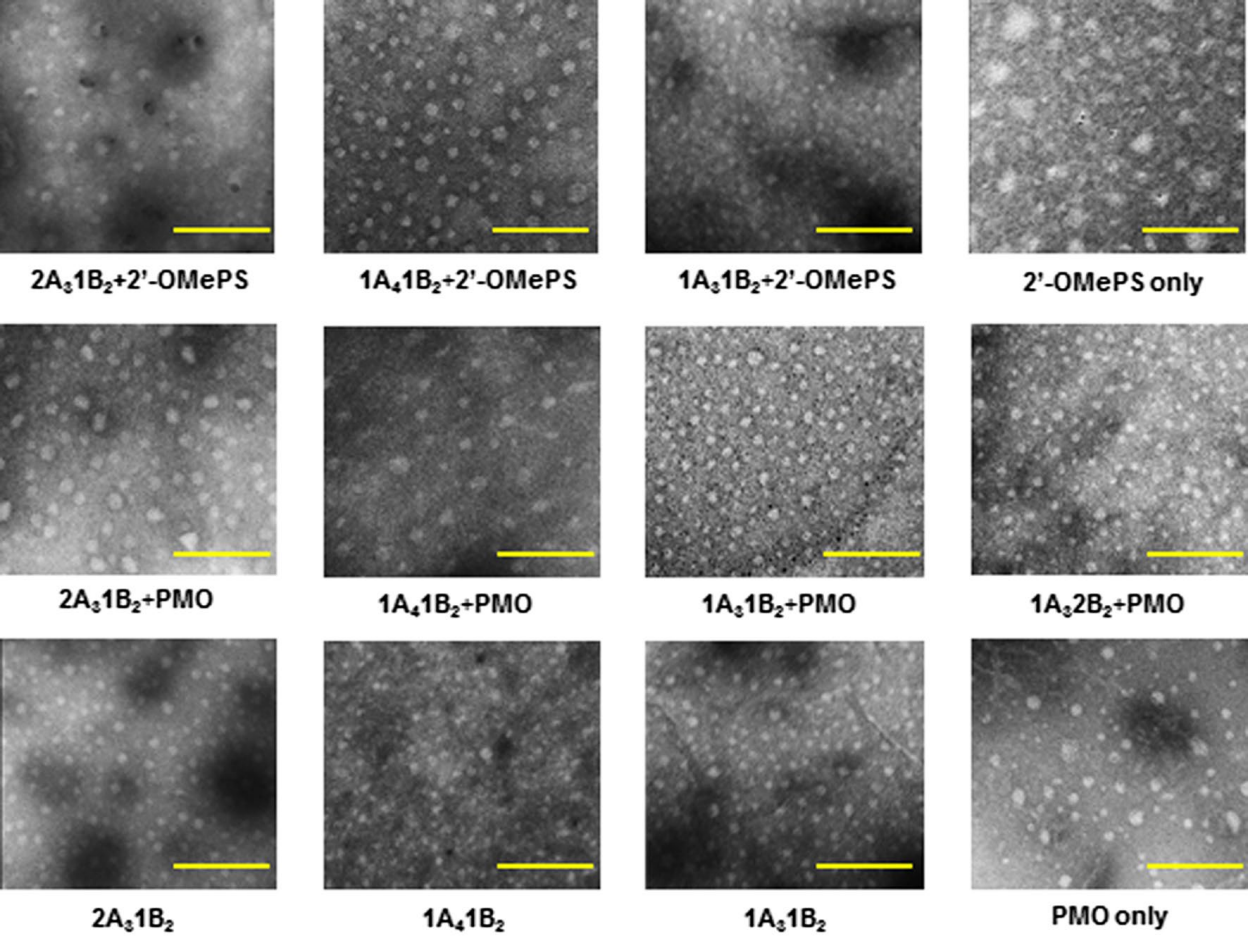
Negatively stained transmission electron micrographs of TAPs, PMO only, and TAPs (10 μg) complexed with PMO (5 μg) or 2′-OMePS (2 μg) (scale bar 100 nm)

To better understanding the delivery action of polymer-mediated AOs, we exemplified the TAP polymers: 1A_3_1B_2_, 1A_3_2B_2_, 2A_3_1B_2_ and 1A_4_1B_2_, and corresponding TAP/AO polyplexes (TAP/PMO = 10/5; TAP/2′-OMePS = 10/2 based on the in vitro results) examined intuitively under transmission electron microscopy (TEM). The followings are illustrated in Fig. 5: (1) The polymer TAPs alone formed smaller particles via self-assembly with different size either more hydrophobic (2A_3_1B_2_) or more hydrophilic (1A_4_1B_2_) likely due to its amphiphile composition. (2) The PMO oligonucleotides alone formed particles with the size below 50 nm, that is most likely a result of hydrophobic interactions among PMO molecules; while the 2′-OMePS only formed different size particles due to the aggregation. (3) The polyplex of TAP/PMO at weight ratio of 10/5 formed spherical particles larger than the corresponding TAP alone with an average diameter around 40–70 nm, that is mainly due to the hydrophobic interaction and hydrogen-bonds between the PMO and polymer. (4) The TAP/2′-OMePS at weight ratio of 10/2 polyplex gave slightly smaller particles compared with TAP/PMO probably due to the additional charge–charge interaction. (5) The more hydrophobic 1A_3_1B_2_/AO complex showed smaller particle size than the more hydrophilic 1A_4_1B_2_/AO complex one. (6) The more positive 1A_3_2B_2_/AO complex gave smaller or condensed particle compared with the less positive 1A_3_1B_2_/AO complex. These results coincident with our previous reported that the more hydrophobic and more cationic polymers achieved more condensed nanoparticles with oligonucleotides [30–35]. The interaction between polymer and oligonucleotides is important to affect their delivery performances into cells or tissues, the nanosized polyplex particles should help to overcome biological barriers, increase cellular uptake, and enhance escape from membrane compartments [36–38].

### Delivery of AOs with TAPs in vivo

#### Local delivery

In view of the above results that all TAPs showed improved delivery performance for AO exon-skipping, accompanied by much lower cytotoxicity compared with PEIs in vitro, we firstly evaluated them in vivo via intramuscular (i.m.) injection of AOE23 formulated with TAP polymers to tibialis anterior (TA) muscles of *mdx* mice (aged 4–5 weeks). The *mdx* mouse contains a nonsense mutation in exon 23 and prevents production of a functional dystrophin protein, whereas targeted removal of the mutated exon through exon-skipping, can restore the reading frame and expression of dystrophin protein.

#### PMO Delivery

**Fig. 6:**
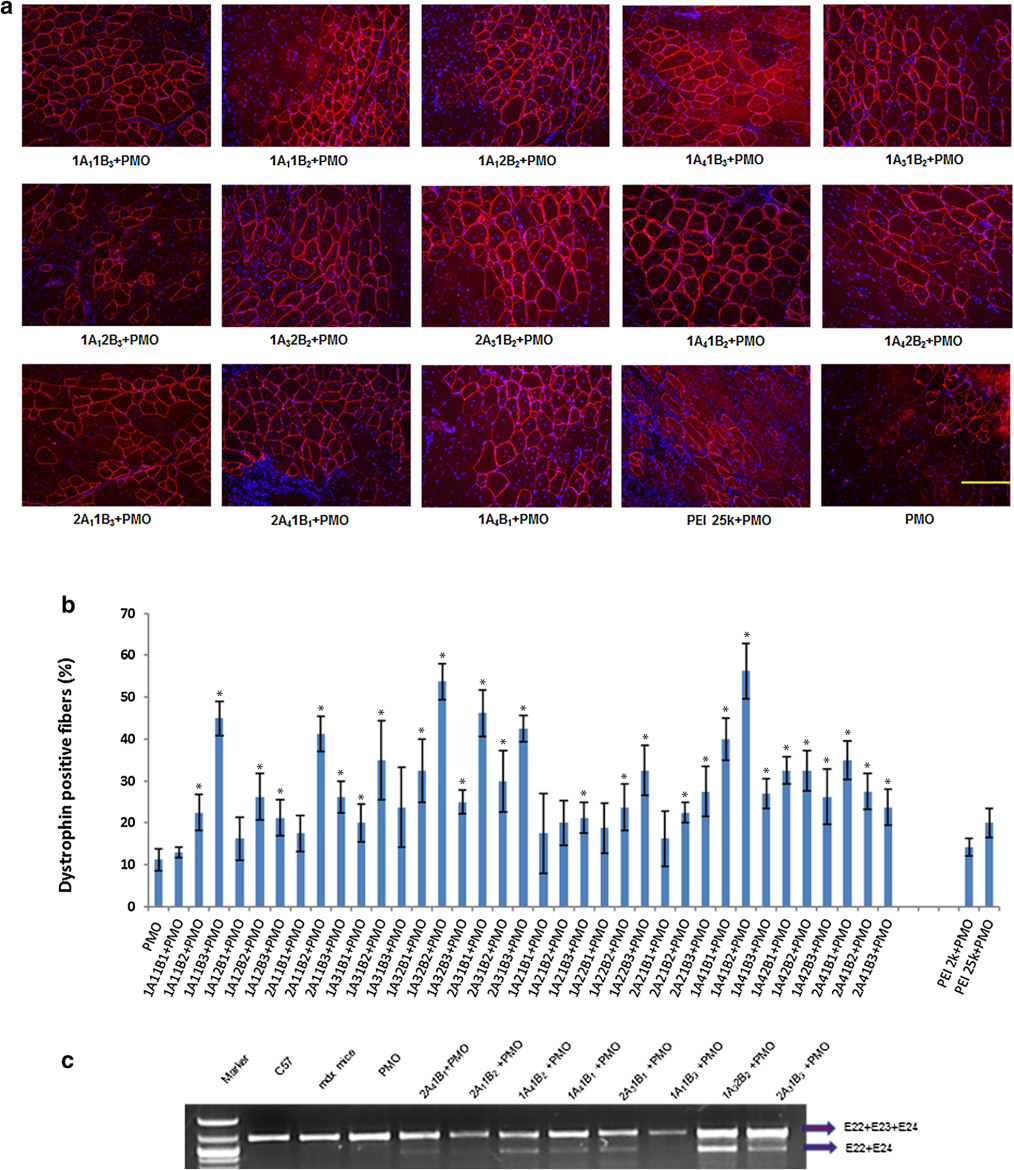
Restoration of dystrophin in tibialis anterior muscles of *mdx* mice (aged 4–5 weeks) 2 weeks after intramuscular injection with 10 μg polymer formulated PMOE23 (2 μg) in 40 μL saline. Muscles treated with PMOE23 only was used as controls. **a** Dystrophin was detected by immunohistochemistry with rabbit polyclonal antibody P7 against dystrophin. Blue nuclear staining with 4,6-diamidino-2-phenylindole (original magnification ×200; scale bar 200 µm). **b** The percentage of dystrophin-positive fibers (mean ± SD, n = 5, Two-tailed t-test, *p ≤ 0.05 compared with PMO). **c** Detection of exon 23 skipping by RT-PCR. Total RNA of 100 ng from each sample was used for amplification of dystrophin mRNA from exon 20 to exon 26. The upper bands (indicated by E22 + E23 + E24) correspond to the normal mRNA, and the lower bands (indicated by E22 + E24) correspond to the mRNA with exon E23 skipped

Each TA muscle received 2 µg PMOE23 formulated with 10 µg TAP polymer in 40 µL saline. The treated muscles were harvested 2 weeks after injection as our previous study [30–35]. Immunohistochemistry showed that PEI 2.0k, PEI 25k formulated PMO and PMO alone induced around 13%, 20%, and 11% dystrophin positive fibers in one cross-section of the TA muscle, respectively. Dystrophin positive fibers increased dramatically in the muscles treated with TAP-formulated PMOE23. Such as, the dystrophin positive fibers being 41%, 35%, 54%, 46%, 40%, 56%, 45% with the use of 2A_1_1B_2_, 1A_3_1B_2_, 1A_3_2B_2_, 2A_3_1B_1_,1A_4_1B_1_, 1A_4_1B_2_, 1A_1_1B_3_ formulated PMO, respectively. Most significantly, the use of 1A_3_2B_2_, 1A_4_1B_2_ formulated PMO reached up approximately fivefold of PMO alone (Fig. 6). The levels of exon-skipping confirmed with RT-PCR were 24.4%, 28.6%, 47.2%, 28.0%, 34.1%, 35.0%, 55.7%, 38.2% and 13.8% for 2A_4_1B_1_, 2A_1_1B_2,_ 1A_4_1B_2_, 1A_4_1B_1_, 2A_3_1B_1_, 1A_1_1B_3_, 1A_3_2B_2_, 2A_3_1B_3_ formulated PMO and PMO only, respectively. The exon-skipped level is consistent with the visualized positive fibers, and further demonstrated that the amphiphilic nature (either more hydrophilic A_2_/A_4_-constructed TAPs or more lipophilic A_1_/A_3_-constructed TAPs) is crucial for the delivery of neutral-charged oligonucleotides PMO both in vitro and in vivo.

#### 2′-OMePS delivery

**Fig. 7:**
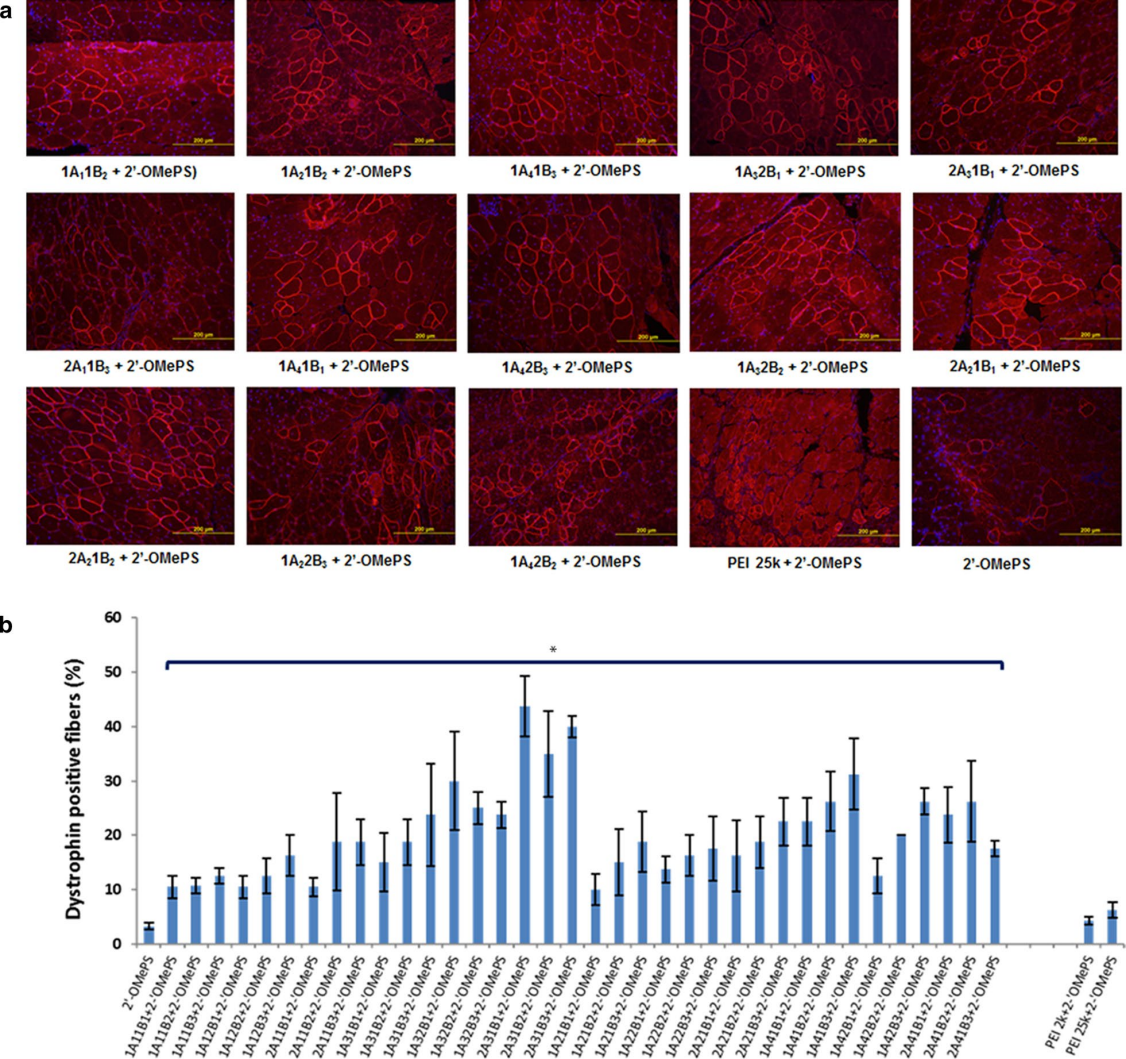
Restoration of dystrophin in tibialis anterior muscles of *mdx* mice (aged 4–5 weeks) 2 weeks after intramuscular injection with 20 μg polymer formulated 2′-OMePS (5 μg) in 40 μL saline. Muscles treated with 2′-OMePS only was used as controls. **a** Dystrophin was detected by immunohistochemistry with rabbit polyclonal antibody P7 against dystrophin. Blue nuclear staining with 4, 6-diamidino-2-phenylindole (original magnification ×200; scale bar 200 μm). **b** The percentage of dystrophin-positive fibers (mean ± SD, n = 5, Two-tailed t-test, *p ≤ 0.05 compared with 2′-OMePS)

Next, we evaluated the effects of the TAPs for negative-charged 2′-OMePSE23 delivery in vivo by i.m. injection. All TAPs were examined at the dose of 20 µg mixed with 5 µg 2′-OMePSE23 in 40 µL saline, and the treated TA muscles were harvested 2 weeks after injection also. Immunohistochemistry showed that the numbers of dystrophin-positive fibers increased up to 3- to 15-folds in the muscles treated with same amount of 2′-OMePS formulated with TAP polymers. Particularly, dystrophin-positive fibers were induced to 30%, 44%, 35%, 40% and 32% with 1A_3_2B_1_, 2A_3_1B_1_, 2A_3_1B_2_, 2A_3_1B_3_, 1A_4_1B_3_ formulation, respectively. In contrast, PEI 25k-mediated 2′-OMePS produced around 6% positive fibers (Fig. 7). On one hand, exceptionally, the 2A_3_1B series increased dystrophin induction more effectively for 2′-OMePSE23 delivery than other TAPs, probably due to its condensed particles and more favorable positive surface charge of TAP/2′-OMePS polyplex, as demonstrated by TEM morphology. On the other hand, both more hydrophilic A_2_/A_4_-constructed TAPs and more lipophilic A_1_/A_3_-constructed TAPs showed effective in vivo when compared with that only more hydrophilic A_1_/A_3_-constructed TAPs working in vitro. Furthermore, it indicates that there is gap in delivery performance between in vitro and in vivo. Overall, the delivery efficiency of 2′-OMePS is lower than that of PMO, the reason is that the charge-neutral PMO probably has less impediment to cell-surface contact and may thus enter muscle fibers more effectively, particularly those with leaky membrane in the dystrophic muscles [42, 43].

#### Systemic delivery

**Fig. 8:**
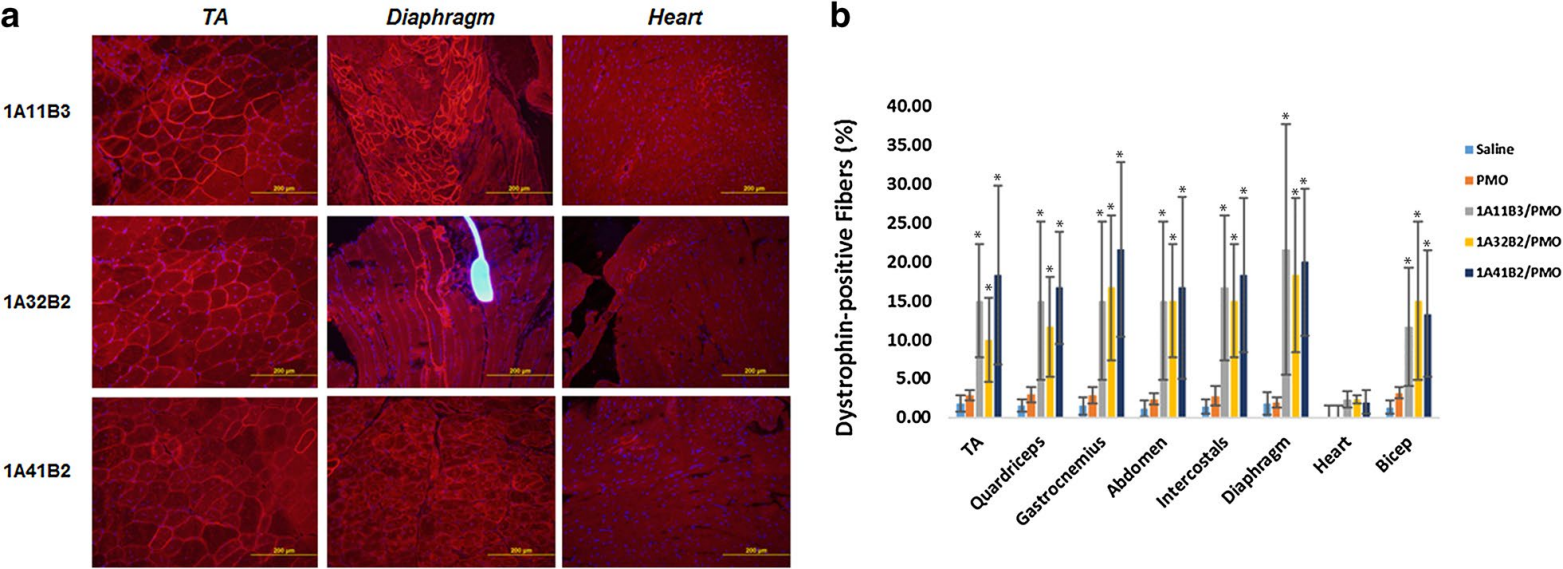
Dystrophin expression in different muscles and serum study of mdx mice (aged 4–5 weeks) 2 weeks after systemic administration of PMO with TAPs. Each mouse was injected with 1 mg PMOE23 with and without TAP (0.5 mg). **a** Immunohistochemistry with antibody P7 for the detection of dystrophin (original magnification ×100; scale bar 200 µm). **b** Percentage of dystrophin-positive fibers in different muscle tissues (mean ± SD, n = 5, Two-tailed t-test, *p ≤ 0.05 compared with 1 mg PMO)

DMD—a systemic disease, affects body-wide muscles including cardiac muscle, therefore systemic treatment is indispensable. Based on the results in vivo locally, 1A_1_1B_3_,1A_3_2B_2_ and 1A_4_1B_2_ were further evaluated their effects for PMO systemic delivery by intravenous (i.v.) injection at the dose of 0.5 mg formulated with 1 mg PMOE23 (Fig. 8). The control PMOE23 alone induced dystrophin expression in less than 3% of muscle fibers in all skeletal muscles and no detectable dystrophin in cardiac muscle 2 weeks after injection. PMOE23 formulated with TAPs produced dystrophin positive fibers 10–22% in skeletal muscles, with the highest levels around 22% with 1A_4_1B_2_ in gastrocnemius muscle and over 20% with 1A_1_1B_3_ in diaphragm muscle, respectively. Importantly, immunohistochemistry demonstrated membrane-localized dystrophin in about 1–3% of cardiac muscle fibers in some areas of the heart treated with the single dose of TAPs. Additionally, the low levels of dystrophin induction in cardiac muscle could be beneficial to the patients, against only occasional one or two positive fibers were observed in cardiac tissue in the mice treated with PMO alone [7, 16].

No signs of abnormal behavior or change in body weight and overall condition were observed during treatment with any TAP/AO polyplex in the mice treated both local and systemic delivery. No pathologic changes of the liver, kidney and lung of the treated mice were detected by Hematoxylin and Eosin (H&E) staining, confirming their low tissue toxicity as demonstrated previously in pDNA delivery [34]. The results suggest that TAPs could be further explored for potential antisense delivery to increase exon-skipping efficiency especially for the treatment of muscular dystrophies.

## Conclusions

The study of triazine-cored amphiphilic polymers for AO delivery in vitro and in *mdx* mice demonstrated that the carrier’s performances are related to the molecular size, compositions and hydrophilic-lipophilic balance (HLB) of the polymers, as well as the AO’s chemical structure. In general, the polymers with more lipophilic and higher PEI contents are suitable for negative charged 2′-OMePS delivery; meanwhile, amphiphilic nature is the key as uncharged PMO delivery carrier, though it’s hard to conclude the performance correlation between in vitro and in vivo. The variability of individual TAP polymers for delivery of negatively charged pDNA, 2′-OMePS and uncharged PMO highlighted the complexity of the interaction between polymer and the therapeutic agents, the difference in the delivery mechanism. The unique hydrophobic interaction between the TAP polymer and AO creates a more stable complex in primarily hydrophilic environments and further enhances complex-plasma membrane interactions both in vitro and in vivo. For the conventional delivery system, all the TAPs have lower toxicity as compared with Endoporter or PEI 25K. Improved exon-skipping efficiency was observed in vitro with all TAP polymers and some of them exhibit comparable to or higher than positive control. The in vivo study in *mdx* mice demonstrated significantly enhanced exon-skipping of AO with the TAP polymers compared with AOs alone.

## Supplementary information

**Additional file 1.** 1) Polymer’s structure and corresponding code; 2) Comparison of transduction and cytotoxicity between TAP (10 μg) and LF-2k (4 μg) mediated 2′-OMePSE50.

## Data Availability

All data generated or analyzed during this study are included in this article and in Additional file 1.

## Abbreviations

TAPs: Triazine-cored amphiphilic polymers
DMD: Duchenne muscular dystrophy
AO: Antisense oligonucleotides
2′-OMePS: 2′-*O*-Methyl phosphorothioate RNA
PMO: Phosphorodiamidate morpholino oligomers
HLB: Hydrophilic-lipophilic balance
TEM: Transmission electron microscopy
MTS: 3-(4,5-Dimethylthiazol-2-yl)-5-(3-carboxymethoxyphenyl)-2-(4-sulfophenyl)-2*H*-tetrazolium
LF-2k: Lipofectamine-2000
PEI: Polyethyleneimine
DMEM: Dulbecco’s modified Eagle’s medium
FBS: Fetal bovine serum
HEPES: 4-(2-Hydroxyethyl)-1-piperazineethanesulfonic acid
