# Supplementary material for: Triazine-cored polymeric vectors for antisense oligonucleotide delivery in vitro and in vivo
Source: J Nanobiotechnology. 2020 Feb 18;18:34. doi: 10.1186/s12951-020-0586-8 (PMC7029474; doi:10.1186/s12951-020-0586-8)
Supplement: Supplementary file 1 — Additional file 1. 1) Polymer’s structure and corresponding code; 2) Comparison of transduction and cytotoxicity between TAP (10 μg) and LF-2k (4 μg) mediated 2′-OMePSE50. [file 12951_2020_586_MOESM1_ESM.docx]

**Additional file 1.**

**Triazine-cored Polymeric Vectors for Antisense Oligonucleotide Delivery *in vitro* and *in vivo***

Mingxing Wang*, Bo Wu, Jason D Tucker, Sapana N Shah, Peijuan Lu, Qilong Lu

McColl^-^Lockwood Laboratory for Muscular Dystrophy Research, Carolinas Medical Center, 1000 Blythe Blvd. Charlotte, NC 28231, USA

Phone: 1-704-355-5588;  Fax: 1-704-355-1679; Email: mingxing.wang@atriumhealth.org

**Synthesis and characterization of Triazine-cored amphiphilic polymers (TAPs)**

The synthesis and characterization of TAPs have been reported in our previous study.^30^ The structure and code of Jeffamine M series and LPEI were given in Figure S1, and the code abbreviations and corresponding compositions of the TAP polymers are presented in Table S1.


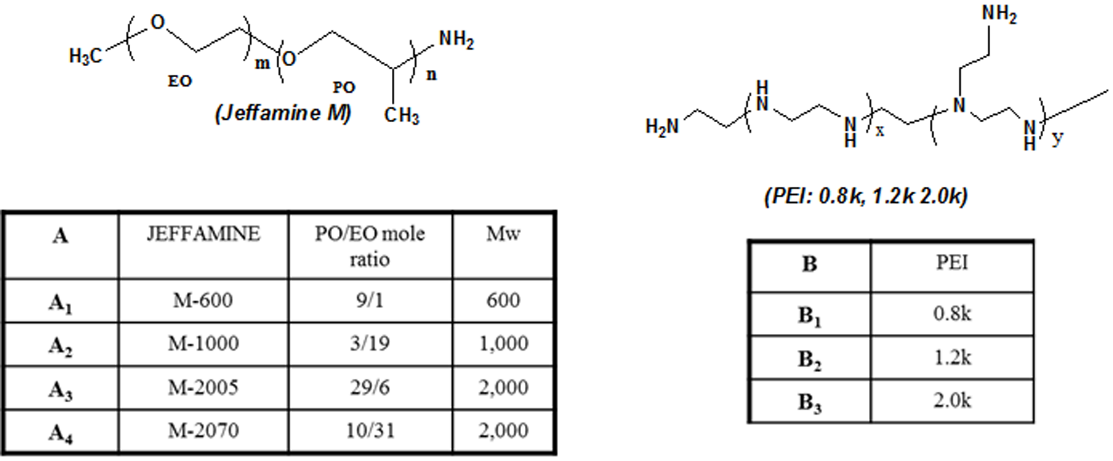


**Figure S1. Structure illustration of substituents A (Jeffamine M ) and B (LPEI).**

**Table S1. Polymer code abbreviations and corresponding compositions**

| **Code** | **Composition** | **Code** | **Composition** | **Code** | **Composition** | **Code** | **Composition** |
| --- | --- | --- | --- | --- | --- | --- | --- |
| 1A_4_1B_1_ | M2070-Cy(Cl)-PEI 0.8k | 1A_3_1B_1_ | M2005-Cy(Cl)-PEI 0.8k | 1A_2_1B_1_ | M1000-Cy(Cl)-PEI 0.8k | 1A_1_1B_1_ | M600-Cy(Cl)-PEI 0.8k |
| 1A_4_1B_2_ | M2070-Cy(Cl)-PEI 1.2k | 1A_3_1B_2_ | M2005-Cy(Cl)-PEI 1.2k | 1A_2_1B_2_ | M1000-Cy(Cl)-PEI 1.2k | 1A_1_1B_2_ | M600-Cy(Cl)-PEI 1.2k |
| 1A_4_1B_3_ | M2070-Cy(Cl)-PEI 2.0k | 1A_3_1B_3_ | M2005-Cy(Cl)-PEI 2.0k | 1A_2_1B_3_ | M1000-Cy(Cl)-PEI 2.0k | 1A_1_1B_3_ | M600-Cy(Cl)-PEI 2.0k |
| 1A_4_2B_1_ | M2070-Cy-2PEI 0.8k | 1A_3_2B_1_ | M2005-Cy-2PEI 0.8k | 1A_2_2B_1_ | M1000-Cy-2PEI 0.8k | 1A_1_2B_1_ | M600-Cy-2PEI 0.8k |
| 1A_4_2B_2_ | M2070-Cy-2PEI 1.2k | 1A_3_2B_2_ | M2005-Cy-2PEI 1.2k | 1A_2_2B_2_ | M1000-Cy-2PEI 1.2k | 1A_1_2B_2_ | M600-Cy-2PEI 1.2k |
| 1A_4_2B_3_ | M2070-Cy-2PEI 2.0k | 1A_3_2B_3_ | M2005-Cy-2PEI 2.0k | 1A_2_2B_3_ | M1000-Cy-2PEI 2.0k | 1A_1_2B_3_ | M600-Cy-2PEI 2.0k |
| 2A_4_1B_1_ | 2M2070-Cy-PEI 0.8k | 2A_3_1B_1_ | 2M2005-Cy-PEI 0.8k | 2A_2_1B_1_ | 2M1000-Cy-PEI 0.8k | 2A_1_1B_1_ | 2M600-Cy-PEI 0.8k |
| 2A_4_1B_2_ | 2M2070-Cy-PEI 1.2k | 2A_3_1B_2_ | 2M2005-Cy-PEI 1.2k | 2A_2_1B_2_ | 2M1000-Cy-PEI 1.2k | 2A_1_1B_2_ | 2M600-Cy-PEI 1.2k |
| 2A_4_1B_3_ | 2M2070-Cy-PEI 2.0k | 2A_3_1B_3_ | 2M2005-Cy-PEI 2.0k | 2A_2_1B_3_ | 2M1000-Cy-PEI 2.0k | 2A_1_1B_3_ | 2M600-Cy-PEI 2.0k |


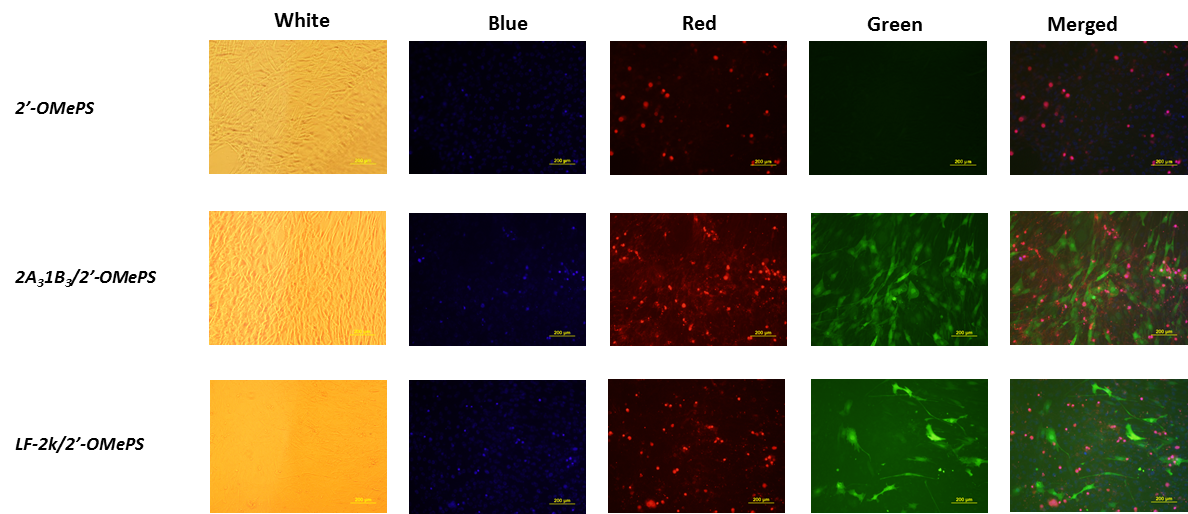


**Figure S2. Comparison of transduction and cytotoxicity between TAP (10 µg) and LF-2k (4 µg) mediated 2′-OMePSE50 (2 µg) stained by Propidium iodide (PI, red) and Hoechst 33342 (blue) after six-day delivery (original magnification, x200; scale bar: 500 µm).**
